# The pro-apoptotic function of the *C. elegans* BCL-2 homolog CED-9 requires interaction with the APAF-1 homolog CED-4

**DOI:** 10.1126/sciadv.adn0325

**Published:** 2024-10-09

**Authors:** Nolan Tucker, Peter Reddien, Bradley Hersh, Dongyeop Lee, Mona H. X. Liu, H. Robert Horvitz

**Affiliations:** ^1^ Howard Hughes Medical Institute, Department of Biology, Massachusetts Institute of Technology, Cambridge, MA 02139, USA.; ^2^ Harvard Medical School, Boston, MA 02115, USA.

## Abstract

In *Caenorhabditis elegans*, apoptosis is inhibited by the BCL-2 homolog CED-9. Although canonically anti-apoptotic, CED-9 has a poorly understood pro-apoptotic function. CED-9 is thought to inhibit apoptosis by binding to and inhibiting the pro-apoptotic *C. elegans* APAF-1 homolog CED-4. We show that CED-9 or CED-4 mutations located in their CED-9–CED-4 binding regions reduce apoptosis without affecting the CED-9 anti-apoptotic function. These mutant CED-9 and CED-4 proteins are defective in a CED-9–CED-4 interaction in vitro and in vivo, revealing that the known CED-9–CED-4 interaction is required for the pro-apoptotic but not for the anti-apoptotic function of CED-9. The pro-apoptotic CED-9–CED-4 interaction occurs at mitochondria. In mammals, BCL-2 family members can activate APAF-1 via cytochrome c release from mitochondria. The conserved role of mitochondria in CED-9/BCL-2–dependent CED-4/APAF-1 activation is notable and suggests that understanding how CED-9 promotes apoptosis in *C. elegans* could inform the understanding of mammalian apoptosis and how disruptions of apoptosis promote certain human disorders.

## Teaser

Mutations that perturb interaction between CED-9 and CED-4 disrupt the pro-apoptotic but not the anti-apoptotic function of CED-9.

## INTRODUCTION

Programmed cell death is an evolutionarily conserved process essential for proper development and tissue homeostasis in metazoans (*1*, *2*). In humans, either excessive or insufficient cell death can result in diseases, including cancers and certain autoimmune and neurodegenerative disorders (*3*–*5*). In *Caenorhabditis elegans*, four genes—*egl-1*, *ced-9*, *ced-4*, and *ced-3*—are primarily responsible for the control of apoptotic cell death (*6*–*8*). It has been proposed based on genetic, protein interaction, and localization studies that CED-9 (a homolog of mammalian BCL-2) protects cells from apoptosis by sequestering the pro-apoptotic protein CED-4 (APAF-1 in mammals) to mitochondria (*9*–*11*). According to this model, in cells fated to die, EGL-1 (a BH3-only member of the Bcl-2 superfamily) binds CED-9, causing a conformational change in CED-9 that results in the release of CED-4 (*12*). After its release from CED-9, CED-4 localizes to the perinuclear membrane where it activates the caspase CED-3 (*10*, *11*). A strong *ced-9* loss-of-function mutation leads to maternal-effect lethality, presumably as a consequence of excessive cell death, and animals carrying loss-of-function mutations in both *ced-9* and a downstream cell-death promoting gene—either *ced-4* or *ced-3*—are viable because cell death is prevented. However, and counterintuitively, a loss-of-function mutation in *ced-9* can enhance the partial cell-death defect of animals with a weak loss-of-function mutation in the pro-apoptotic caspase gene *ced-3* (*13*). This finding suggests that, in addition to its anti-apoptotic role, CED-9 has a pro-apoptotic function.

The existence of a pro-apoptotic function of CED-9 implies that the canonical model for *C. elegans* cell death, in which CED-9 functions only as an anti-apoptotic protein, is incomplete. Furthermore, the canonical model proposes that, in living cells, cell death is blocked by the CED-9–dependent sequestration of CED-4 to mitochondria. However, in *ced-9(n1653ts)* temperature-sensitive mutant animals at a permissive temperature, some CED-4 protein is localized to the perinuclear membrane, as assayed by anti–CED-4 polyclonal antibody localization (*9*). The *ced-9(n1653ts) *allele, causes lethality at nonpermissive temperatures [like *ced-9(0)* alleles] but at permissive temperatures allows viability [like *ced-9(+)* and weak *ced-9(lf)* loss-of function alleles]. The observation that CED-4 protein can be localized to the perinuclear membrane without causing lethality suggests that CED-4 localization at the perinuclear membrane is not sufficient to drive apoptosis. In addition, despite the ability of *ced-9(lf)* alleles to enhance ectopic cell survival caused by weak loss-of-function alleles in the cell-death promoting *ced-3* gene, loss of *ced-9* function in the presence of a weak *ced-3* loss-of-function mutation results in CED-4 localization to the perinuclear membrane (*9*). Furthermore, overexpression of a functional CED-4::GFP (green fluorescent protein) transgene fails to induce the death of germ cells in which the CED-4::GFP protein is localized to the perinuclear membrane (*14*), again uncoupling CED-4 localization at the perinuclear membrane from the activation of apoptotic death. In short, these observations suggest that CED-4 localization at the perinuclear membrane is not sufficient to cause cell death.

Here, we report that mutations in the known CED-9–CED-4 binding region cause a loss of the pro-apoptotic but not the anti-apoptotic function of CED-9. In addition, these mutations prevent the CED-9–dependent sequestration of CED-4 to mitochondria. Our results suggest that—opposite to the canonical model for *C. elegans* cell death in which a CED-9–CED-4 interaction (and consequent CED-4 mitochondrial localization) are anti-apoptotic—a CED-9–CED-4 interaction (and possibly CED-4 mitochondrial localization) are required for the pro-apoptotic function of CED-9. Because *C. elegans* cell-death genes show notable homology to mammalian cell-death genes (*13*–*17*), these findings have implications for our understanding of the functions of mammalian BCL-2 family members in cell death and how perturbation of their functions can affect human diseases, including certain neurogenerative disorders and cancers.

## RESULTS

### CED-9 has a pro-apoptotic function

**Fig. 1.**
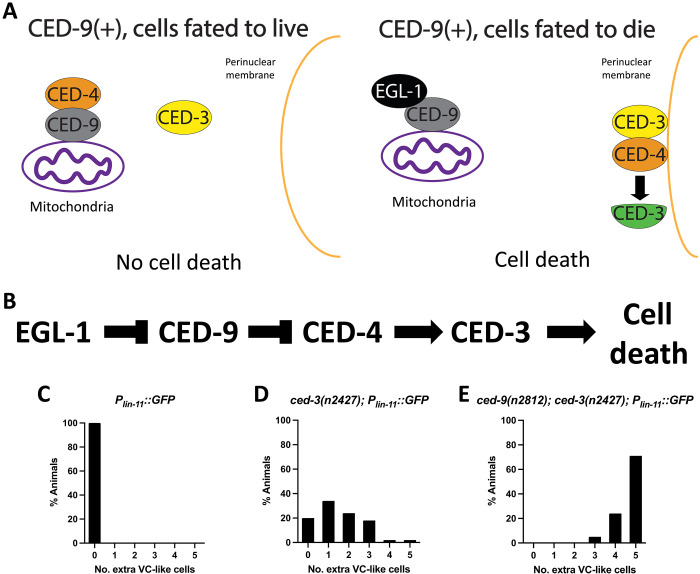
*ced-9* has a noncanonical pro-apoptotic function. (**A**) Canonical model for apoptosis in *C. elegans*. CED-9 acts to prevent cell death by sequestering the pro-apoptotic protein CED-4 to mitochondria. Cells fated to die express the pro-apoptotic EGL-1, which binds CED-9, causing a conformational change in CED-9 that results in the release of CED-4 from mitochondria. After its release from CED-9, CED-4 localizes to the perinuclear membrane, where it activates the caspase CED-3, thus inducing cell death. (**B**) Canonical genetic pathway for apoptosis in *C. elegans*. (**C** to **E**) Defects in cell killing, as assayed using the transgene *nIs106[P_lin-11_::GFP]*, a GFP reporter expressed in both the VC motor neurons and “extra” VC-like undead cells. (C) In wild-type animals, this transgene detects no extra VC-like cells (*n* = 50). (D) *ced-3(n2427)* causes the ectopic survival of ~1.7 extra VC-like cells (*n* = 50). (E) The null allele *ced-9(n2812)* enhances the cell-killing defect caused by *ced-3(n2427)* from ~1.7 to ~4.7 extra VC-like cells (*n* = 50).

In the canonical model of *C. elegans* apoptosis, the anti-apoptotic protein CED-9 prevents apoptosis by sequestering the pro-apoptotic CED-4 protein to mitochondria (Fig. 1, A and B). *ced-9(lf)* mutations cause maternal-effect lethality, presumably via excessive apoptosis because mutations in the downstream pro-apoptotic genes *ced-3* and *ced-4* suppress this lethality. However, *ced-9(lf)* mutations can also enhance the cell-death defect caused by a weak loss-of-function mutation in either *ced-3* or *ced-4* (*13*), indicating the existence of a poorly understood pro-apoptotic role for *ced-9*. One such enhancement of a cell-death defect by *ced-9(lf)* mutations involves the ventral cord (VC) neurons in the ventral nervous system. In wild-type (WT) animals, six VC neurons (P3-8.aap) survive and express the GFP reporter *nIs106[P_lin-11_::GFP]*, whereas six VC homologs (P1-2,9-12.aap) undergo apoptosis (*18*). In mutants carrying a null allele of either *ced-3* or *ced-4*, the six VC homologs that normally die instead survive, differentiate, and express characteristics of the VC neurons (*19*), including expression of the *P_lin-11_::GFP* reporter; five of these six “undead” VC-like cells can be reliably scored using this GFP reporter. Whereas *ced-3* null mutants show ectopic survival of about 5 extra cells, animals carrying a weak loss-of-function allele of *ced-3*, *ced-3(n2427)*, show ectopic survival of about 1.7 extra VC-like cells (Fig. 1, C and D) (*20*). By contrast, when *ced-3(n2427)* is paired with the *ced-9* null allele *ced-9(n2812)*, these double-mutant animals show roughly 4.7 extra VC-like cells (Fig. 1E). This result indicates that a lack of *ced-9* function can cause a notable increase in VC cell survival, indicative of the poorly understood pro-apoptotic function of *ced-9*.

### An EMS screen for *ced-3(lf)* enhancers generated CED-9–CED-4 binding region mutations

**Fig. 2.**
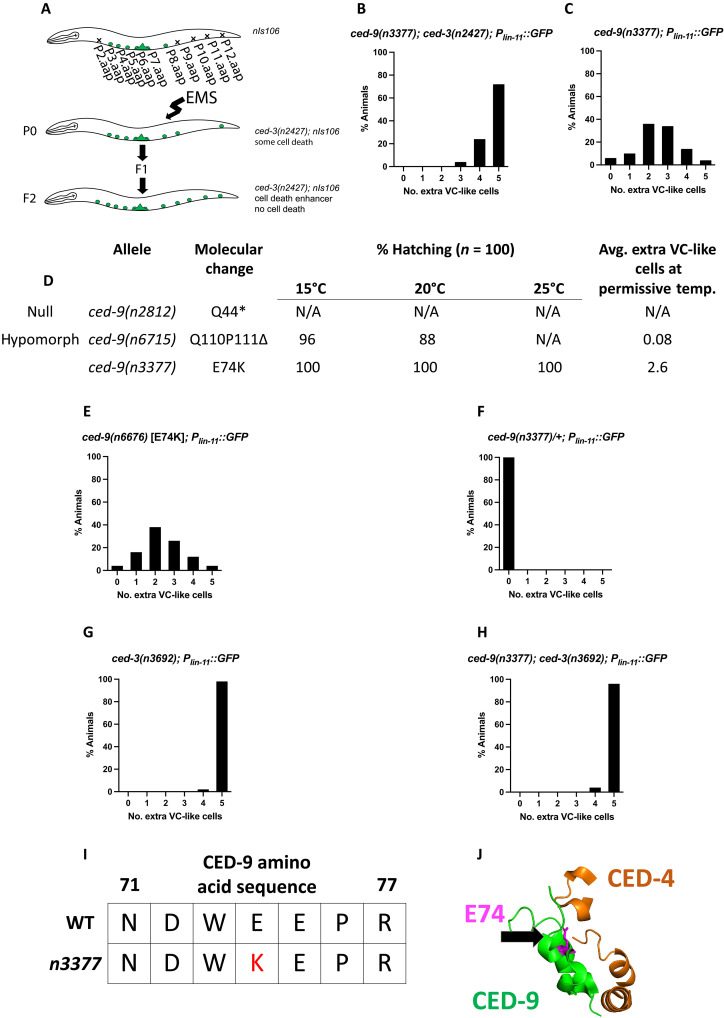
An EMS screen for enhancers of *ced-3(n2427)* isolated a mutation that disrupts the CED-4 binding region of CED-9. (**A**) An EMS screen for enhancers of the cell-death defect mediated by a weak loss-of-function allele of *ced-3*, *ced-3(n2427)* (*20*). (**B** and **C**) This screen identified *ced-9(n3377)*, which is homozygous viable, enhances the cell-killing defect caused by *ced-3(n2427)* and results in the ectopic survival of ~2.6 extra VC-like cells on its own (*n* = 50). (**D**) The *ced-9* null allele, *ced-9(n2812)*, does not produce viable progeny at 15°, 20°, and 25°C. A partial loss-of-function allele of *ced-9*, *ced-9(n6715)*, shows some maternal-effect lethality at 15° and 20°C and fails to produce viable progeny at 25°C; this allele causes the ectopic survival of 0.08 VC-like cells on average at 20°C. A total of 100% of eggs laid by *ced-9(n3377)* hatch at 15°, 20°, and 25°C, and *ced-9(n3377)* animals have ~2.6 ectopically surviving VC-like cells on average at 20°C. N/A, not applicable. (**E**) *n3377* was recreated in a strain free from background mutations caused by EMS to rule out the possibility that background mutations caused by EMS cause the loss of cell-killing phenotype (*n* = 50). (**F**) The cell-killing defect caused by *n3377* is recessive (n = 50). (**G** and **H**) The *ced-3* null allele *n3692* is not enhanced by *ced-9(n3377)*, indicating that *ced-9(n3377)* causes extra VC-like cells by blocking apoptosis (*n* = 50). (**I** and **J**) *ced-9(n3377)* carries a missense mutation (E74K) in the CED-9–CED-4 binding region as defined by crystallographic studies (*21*).

In an earlier work, we described anethyl methanesulfonate (EMS) screen for enhancers of the cell-killing defect caused by *ced-3(n2427)* (Fig. 2A) (*20*). In addition to those mutations previously reported, this screen generated an atypical allele of *ced-9*, *ced-9(n3377)*. Like most *ced-9(lf)* alleles, such as the null allele *ced-9(n2812)*, *ced-9(n3377)* enhanced the cell-killing phenotype caused by *ced-3(n2427)* and did so to an extent similar to that caused by a *ced-9* null allele (Figs. 1E and 2B). By contrast, unlike typical *ced-9(lf)* alleles, which on their own cause maternal-effect recessive lethality because of excessive cell death (*7*), *ced-9(n3377)* when crossed into a *ced-3(+)* background was viable and caused a decrease in cell death: In *ced-3(+); ced-9(n3377)* animals, about 2.6 extra VC-like cells survived, in contrast to 0 extra VC-like cells in *ced-3(+); ced-9(+)* animals (Figs. 1C and 2C). Thus, *ced-9(n3377)* mutants lack the maternal-effect lethal (excess cell death) phenotype characteristic of most loss-of-function alleles of *ced-9*, such as *ced-9(n2812)* (Fig. 2D) and hence retain the anti-apoptotic function of *ced-9*. To preclude background mutations as the cause of this atypical phenotype of *ced-9(n3377)* animals, we used CRISPR to independently generate the *n3377* (E74K) *ced-9* mutation. The recreated allele, *ced-9(n6676)*, resulted in the same phenotype as did *ced-9(n3377)* (Fig. 2E), confirming that *n3377* caused the atypical phenotype.

The cell-death defect of *ced-9(n3377)* animals is recessive: Like *ced-9(+)/ced-9(+)* animals, *n3377/+* animals had 0 extra VC-like cells (Fig. 2F). This observation indicates that the *ced-9(n3377)* mutation causes a decrease in the pro-apoptotic function of *ced-9* rather than an increase in the cell-death inhibitory function of *ced-9*. We next confirmed that *ced-9(n3377)* results in ectopic VC-like cells by perturbing cell death rather than a process other than cell death. Specifically, *ced-9(n3377)* failed to enhance the ectopic VC-like cell survival phenotype of animals carrying *ced-3(n3692)*, a null allele of *ced-3* that prevents cell death altogether (Fig. 2, G and H). We conclude that *n3377* likely causes a loss of the cell-death promoting function of *ced-9*. In short, because *ced-9(n3377)* animals showed a defect in executing cell death but did not show maternal-effect lethality, *ced-9(n3377)* animals seem to lack the pro-apoptotic but retain the anti-apoptotic function of *ced-9*.

The *ced-9(n3377)* missense mutation E74K is located in a domain of CED-9 thought to be involved in binding CED-4, based on the crystal structure of a CED-9–CED-4 complex (Fig. 2, I and J) (*21*). The location and nature of the E74K mutation indicate that *n3377* likely disrupts CED-4 binding. Given the cell-killing defect of *ced-9(n3377)* mutants, one possibility is that the pro-apoptotic function of *ced-9* depends on an interaction with CED-4. This hypothesis contradicts the canonical model for *C. elegans* apoptosis, according to which a loss of CED-9–CED-4 interaction should cause widespread ectopic apoptosis and maternal-effect lethality.

**Fig. 3.**
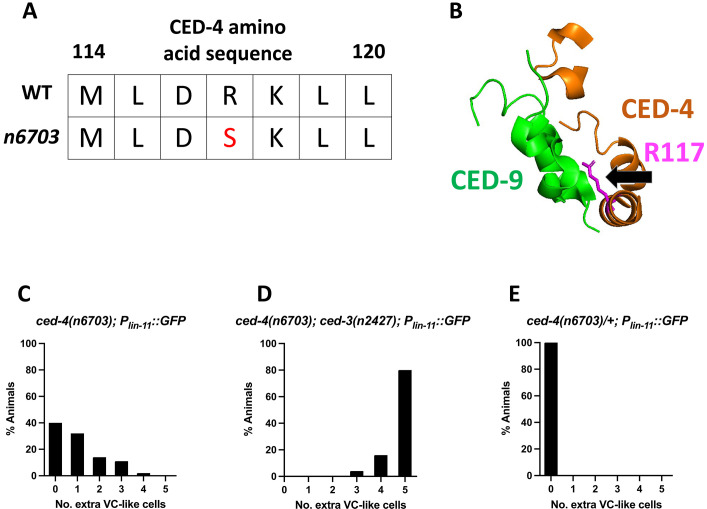
*ced-4(n6703)* causes ectopic VC-like cell survival and contains a mutation in the CED-9 binding region. (**A** and **B**) CED-4[R117S] is a missense mutation in the CED-9 binding region of CED-4, as defined by crystallographic studies (*21*). (**C** to **E**) *ced-4(n6703)* enhances the cell-killing defect caused by *ced-3(n2427)* and results in a recessive cell-death defect that causes ~1.0 extra VC-like cells on its own (*n* = 50).

In addition to *ced-9(n3377)*, the screen described above also generated the *ced-4* allele *n3392*, which is an R117S missense mutation. We independently generated this mutation using CRISPR in a strain free from background mutations caused by EMS and named this allele *ced-4(n6703)* (see Methods). This *ced-4* allele was of interest because R117S is located in the presumptive CED-9 binding region of CED-4 (Fig. 3, A and B) and is therefore likely to disrupt a CED-9–CED-4 interaction. *ced-4(n6703)* animals were viable and displayed a weak cell-killing defect: An average of 1.0 extra VC-like cells were present in these animals, as opposed to the 4.9 extra VC-like cells present in *ced-4(n1162)* null mutant animals (Fig. 3, C and D and Table 1). Both *ced-9(n3377)* and *ced-4(n6703)* also had extra M4-like and RIM/RIC-like cells (table S1), indicating that the cell-killing defect is not limited to VC-like cells. *ced-4(n6703)* did not suppress the maternal-effect lethal phenotype caused by the strong loss-of-function allele *ced-9(n1950 n2161)*. In this way, *ced-4(n6703)* is similar to a wild-type *ced-4(+)* allele and unlike a *ced-4(0)* null allele, e.g., *n1162* (Table 1). These observations are consistent with the hypothesis that *n6703* causes a partial loss of *ced-4* function. However, (i) *ced-4(n6703)* mutants displayed a greater cell-killing defect (1.0 extra VC-like cells) than did two distinct weak hypomorphic reduction-of-function *ced-4* mutants, *ced-4(n2879)* and *ced-4(n2860)* (0.02 and 0.44 extra VC-like cells, respectively); and (ii) *ced-4(n6703)* failed to rescue the maternal-effect lethality of *ced-9(n1950 n2161)*, whereas both of the apparently weaker alleles, *ced-4(n2879)* and *ced-4(n2860)*, did so (Table 1). These observations indicate that *ced-4(n6703)* does not cause a slight general reduction in *ced-4* function but rather—just like *ced-9(n3377)*—specifically results in extra VC-like cells while not affecting the maternal-effect lethal phenotype that is caused by a loss of the anti-apoptotic function of *ced-9*. Because *ced-9(n3377)* mutants are likely deficient in their ability to form a CED-9–CED-4 interaction and lack the pro-apoptotic but not the anti-apoptotic function of *ced-9*, we propose that *ced-4(n6703)* mutants lack a function of *ced-4* needed for the pro-apoptotic function of *ced-9*. In short, these observations suggest that *ced-4(n6703)* causes a cell-killing defect because of a lack of a CED-4(R117S) interaction with CED-9 and that CED-4(R117S) is otherwise functional. These results further suggest that perturbing a CED-9–CED-4 interaction by altering a CED-9–CED-4 interaction domain in either CED-9 or CED-4 is sufficient to cause a cell-killing defect.

**Table 1.** *ced-4(n6703)* does not result in a simple reduction in *ced-4* function. *ced-4(n6703)* causes a stronger cell-killing defect than do *ced-4(n2860)* and *ced-4(n2879)*, as shown by VC-like cell survival. However, *ced-4(n2879)* and *ced-4(n2860)* but not *ced-4(n6703)* can rescue the maternal-effect lethality caused by the *ced-9* loss-of-function allele *ced-9(n1950 n2161)* (*n* = 50). The mutation *unc-69(e587)* is present in the background of all listed *ced-4 ced-9(n1950 n2161)* double mutants because this marker is closely linked to *ced-9* and was used to follow *ced-9(n1950 n2161)* during crossing.

*ced-4* allele	Molecular change	Avg. extra VC-like cells (*P*_*lin-11*_*::GFP*)	Rescues *ced*-*9(n1950 n2161)*
Wild-type	–	0	No
*n2879*	E276K	0.02	Yes
*n2860*	E263K	0.44	Yes
*n6703*	R117S	1.0	No
*n3141*	R53K	3.4	Yes
*n1162* (null)	Q80Ochre	4.9	Yes

### Additional mutations in the CED-4 binding region of CED-9 cause a cell-killing defect

**Fig. 4.**
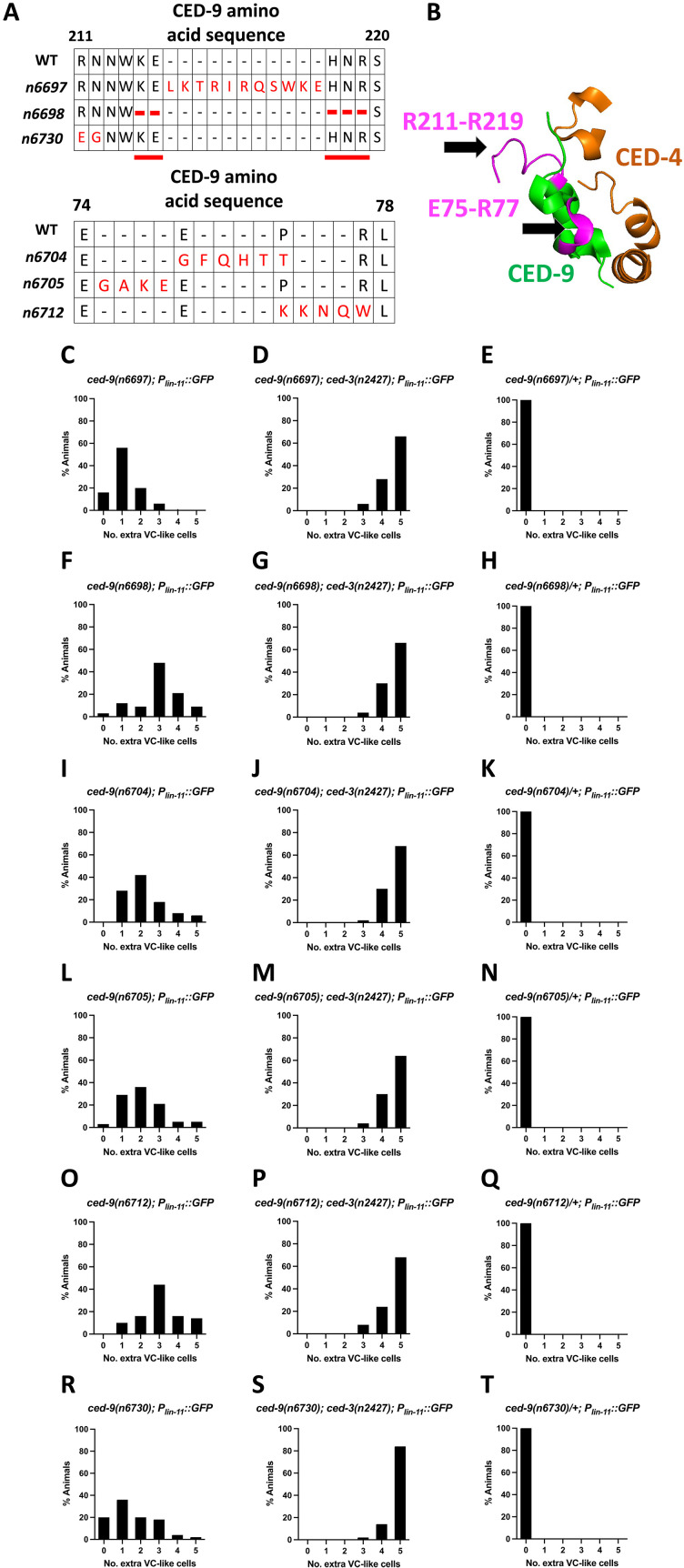
CRISPR-induced mutations in the CED-9–CED-4 binding region of CED-9 result in cell-killing defects. Six alleles of *ced-9* generated by CRISPR-Cas9 carry mutations likely to disrupt the CED-4 binding region. (**A** and **B**) *ced-9(n6697)* carries an 11 amino acid insertion in the CED-4 binding pocket; *ced-9(n6698)* carries a five amino acid deletion in the CED-4 binding pocket; *ced-9(n6730)* carries two missense mutations, R211E and N212G; *ced-9(n6704)* carries a four amino acid insertion in the CED-4 binding pocket as well as two missense mutations, E75G and P76T; *ced-9(n6705)* carries a four amino acid insertion in the CED-4 binding pocket; and *ced-9(n6712)* carries a three amino acid insertion in the CED-4 binding pocket as well as two missense mutations, P76K and R77W. (**C** to **T**) These mutations are viable in a wild-type background, recessively cause ectopic survival of VC-like cells on their own, and enhance the cell-killing defect caused by *ced-3(n2427)* (*n* = 50).

Our observations concerning *ced-9(n3377)* raise the possibility that other *ced-9* mutations affecting a CED-9–CED-4 interaction also specifically disrupt the cell-killing function of *ced-9*. To test this possibility, we used CRISPR to generate additional mutations in the known CED-4 binding region of CED-9 (*21*). We performed these experiments using *ced-3(+)* animals carrying the transgene *nIs106* and screened for worms that that were both viable and showed ectopic VC-like cell survival. This approach generated five alleles of *ced-9*; each of which, like *ced-9(n3377)*, led to viable animals and caused a recessive cell-killing defect and therefore likely maintain the anti-apoptotic but disrupt the pro-apoptotic function of *ced-9*. These alleles—*n6697*, *n6698*, *n6704*, *n6705*, and *n6712—*all contain indels, and in some cases additional missense mutations, in the region encoding the presumptive CED-4–binding pocket and resulted in a cell-killing defect (as assayed by survival of VC-like cells) that was similar in strength to that caused by *ced-9(n3377)* (Fig. 4, A to Q). In addition, we used CRISPR to generate the allele *ced-9(n6730)*, which contains two CED-9 mutations in the presumptive CED-4 binding region, R211E and N212G. CED-9(R211E, N212G) was generated previously by others, expressed in *Escherichia coli*, and shown to be defective in CED-4 binding in vitro (*22*). We found that *ced-9(n6730)* mutants displayed the same phenotype as *n3377*, *n6697*, *n6698*, *n6704*, *n6705*, and *n6712* mutants—viability and a recessive cell-killing defect (Fig. 4, R to T). This observation supports the hypothesis that the phenotype caused by these mutations is a consequence of a disruption in an interaction between CED-9 and CED-4.

These *ced-9* mutations, which are apparently defective in the pro-apoptotic function of *ced-9*, have no apparent effect on the anti-apoptotic function of *ced-9*, as assayed by maternal-effect lethality and ectopic VC cell death (table S2). In short, we identified seven alleles of *ced-9*—as well as one allele of *ced-4*—that prevent the pro-apoptotic function of *ced-9* and disrupt the presumptive CED-9–CED-4 binding region but have no apparent effect on the anti-apoptotic function of *ced-9*. The nature, location, and effects of these mutations suggest that an interaction between CED-9 and CED-4 is required for the pro-apoptotic function of *ced-9* and further suggest that this interaction is not required for the canonical anti-apoptotic function of *ced-9*.

### CED-9–CED-4 binding region mutations prevent mitochondrial CED-4 localization in vivo

**Fig. 5.**
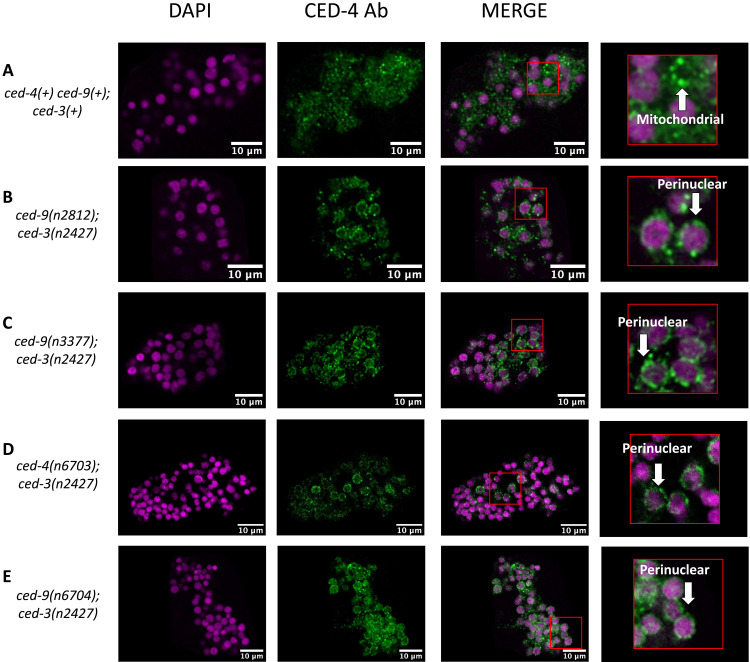
Mutations that disrupt the CED-9–CED-4 binding regions of either CED-9 or CED-4 result in the mislocalization of CED-4 to the perinuclear membrane. (**A**) Mitochondrial localization of CED-4 in wild-type embryos, as shown previously (*9*) (and fig. S3). (**B**) Perinuclear localization of CED-4 in *ced-9(n2812); ced-3(n2427)* embryos; *ced-9(n2812)* is a null allele of *ced-9*. (**C**) Perinuclear localization of CED-4 in *ced-9(n3377); ced-3(n2427)* embryos. (**D**) Perinuclear localization of CED-4 in *ced-4(n6703); ced-4(n2427)* embryos. (**E**) Perinuclear localization of CED-4 in *ced-9(n6704); ced-3(n2427)* embryos. Images were cropped to avoid background noise, and the brightness and contrast of the DAPI and CED-4 channels were adjusted individually using the Fiji software. Ab, antibody.

We next tested if the mutations described above perturb CED-9–dependent sequestration of CED-4 to mitochondria in vivo (*9*). We used a polyclonal antibody against CED-4 previously generated by our laboratory and that in our prior work revealed mitochondrial localization of CED-4 protein in wild-type embryos and perinuclear localization of CED-4 protein in embryos carrying *ced-9(lf)* alleles and a *ced-3* loss-of-function allele (which allowed viability of the strain) (*9*). We confirmed the ability of this antibody to detect CED-4 protein via Western blotting (fig. S1). Using immunofluorescence staining, we also confirmed that wild-type *C. elegans* shows mitochondrial localization of CED-4 (Fig. 5A and fig. S2A) and that embryos carrying the *ced-9* null allele *n2812* and the *ced-3* partial loss-of-function allele *n2427* show perinuclear CED-4 localization (Fig. 5B and fig. S2B). We found that embryos carrying *ced-3(n2427)* and any of the eight CED-9–CED-4 binding region mutant alleles described above—*n3377*, *n6697*, *n6698*, *n6703*, *n6704*, *n6705*, *6712*, and *n6730*—showed perinuclear localization of CED-4 (Fig. 5, C to E, and figs. S2, C to J, and S3, A to E). These observations suggest that these eight mutations, which affect the presumptive CED-9–CED-4 binding regions of CED-4 or CED-9 and cause loss of the pro-apoptotic but not anti-apoptotic function of *ced-9*, are defective in a CED-9–CED-4 interaction required to localize CED-4 to mitochondria.

We examined the possibility that the mutant CED-9–CED-4 interaction alleles described above caused translocation of CED-4 to the perinuclear membrane by disrupting the localization of CED-9 rather than by disrupting a CED-9–CED-4 interaction. CED-4(R117S) embryos that expressed endogenously tagged *wrmScarlet::ced-9*(+) showed CED-9(+) localization to mitochondria as expected (fig. S4). We observed a similar mitochondrial localization of CED-9 in CED-4(+) embryos expressing tagged CED-9(+), CED-9(E74K), or CED-9(R211E, N212G) proteins, indicating that the mutant CED-9–CED-4 interaction alleles described above do not disrupt CED-9 localization.

We also tested a CED-9–CED-4 interaction in vitro. In coimmunoprecipitation experiments, the interactions in vitro of CED-9(E74K) with CED-4(+) and those of CED-9(+) with CED-4(R117S) each were reduced compared to the interaction of CED-9(+) with CED-4(+) (fig. S5).

We conclude that mutations that likely disrupt a CED-9–CED-4 physical interaction defined by the crystal structure (*21*) disrupt an in vivo CED-9–CED-4 interaction that localizes CED-4 to mitochondria. We suggest that the loss of this CED-9–CED-4 interaction and possibly the loss of mitochondrial localization of CED-4 are responsible for the loss of the pro-apoptotic function of *ced-9* in these mutants and that neither this CED-9–CED-4 interaction nor CED-9–dependent mitochondrial sequestration of CED-4 is required for the anti-apoptotic function of *ced-9*.

### CED-9 binding to CED-4 isoforms is unlikely to explain the pro-apoptotic function of *ced-9*

Because a long isoform of CED-4 can prevent cell death when overexpressed (*23*), it is possible that the mutant CED-9–CED-4 interaction alleles described above disrupt the pro-apoptotic but not the anti-apoptotic function of *ced-9* by interfering with the ability of CED-9 to bind to—and inhibit—the minor anti-apoptotic CED-4L long isoform but not the canonically pro-apoptotic CED-4S short isoform. To assess the endogenous functions of CED-4L and CED-4S, we used CRISPR to mutate the endogenous *ced-4* gene and create alleles of *ced-4* that express only CED-4L [*ced-4(n6692)*] or only CED-4S [*ced-4(n6687)*]. We found that a lack of CED-4S caused a cell-death defect (assayed by ectopic VC cell survival) comparable to that of the *ced-4* null allele *n1162* (fig. S6). By contrast, the absence of CED-4L did not cause any detectable ectopic VC cell death or maternal-effect lethality on its own nor did it suppress the VC cell survival phenotype of *ced-9(n3377)* mutants (fig. S6, D to G). On the basis of these data, we are unable to attribute any endogenous function to the CED-4L isoform or relate the two CED-4 isoforms to the CED-9–CED-4 interaction alleles we describe above.

## DISCUSSION

The pro-apoptotic function of the canonically anti-apoptotic *C. elegans* gene *ced-9* has long been mysterious, suggesting a poorly understood fundamental process at the core of the control of apoptotic cell death. We identified seven alleles of *ced-9* and one allele of *ced-4* that disrupt the pro-apoptotic but not the anti-apoptotic function of *ced-9*. All seven *ced-9* alleles contain mutations in the presumptive CED-4 binding region of CED-9, and the *ced-4* allele contains a mutation in the presumptive CED-9 binding region of CED-4. All eight alleles are thus likely to disrupt an interaction between CED-9 and CED-4. We showed that one of these mutant CED-9 proteins, CED-9(E74K), and the mutant CED-4 protein, CED-4(R117S), disrupted the CED-9–CED-4 interaction in vitro; in similar experiments, another of the mutant CED-9 proteins, CED-9(R211E, N212G), was shown by others to disrupt the CED-9–CED-4 interaction in vitro (*22*). We observed that the seven *ced-9* alleles and the one *ced-4* allele all perturbed the CED-9–CED-4 interaction in vivo, as assayed by perinuclear localization of CED-4 in embryos. None of the seven *ced-9* alleles appeared to cause any detectable defect in the anti-apoptotic function of *ced-9*, despite being highly likely to disrupt the known CED-9–CED-4 interaction. Together, our data support a model in which a CED-9–CED-4 interaction is required for the pro-apoptotic function of *ced-9* but is dispensable for its anti-apoptotic function. Such a model has similarities to that for apoptosis in mammals, in which the anti-apoptotic function of BCL-2 (the mammalian homolog of *ced-9*) does not depend on a direct interaction with APAF-1 (the mammalian homolog of *ced-4*) (*24*, *25*). Despite being apparently unable to bind CED-4 (*26*), human BCL-2 expressed in *C. elegans* reduces cell death (*13*, *27*), consistent with a model in which a CED-9–CED-4 interaction is dispensable for the anti-apoptotic role of CED-9 and suggesting that this anti-apoptotic function is evolutionarily conserved because it can be at least partially performed by BCL-2. Such a model contrasts with the canonical model for caspase-mediated apoptosis in *C. elegans* in which a CED-9–CED-4 protein interaction is required for the anti-apoptotic function of *ced-9*. These findings raise important questions for future inquiry.

First, how does a CED-9–CED-4 interaction promote apoptosis? Possible mechanisms could involve a local binding partner of CED-4, a conformational change or chemical modification of CED-4, or—given the mitochondrial localization of CED-9 and CED-4 in wild-type cells (*9*)—a mitochondrial process that is affected by a CED-9–CED-4 complex. Second, what is the anti-apoptotic function of *ced-9*? The uncoupling of the anti-apoptotic role of *ced-9* from CED-4 mitochondrial localization in CED-9–CED-4 interaction mutants indicates that CED-9 inhibits some process other than CED-4 perinuclear localization. Third, what is the pro-apoptotic event triggered by EGL-1 interaction with CED-9 in cells fated to die if this event is not disruption of the CED-9–CED-4 interaction? Whereas cell death is reduced when a CED-9–CED-4 interaction is perturbed, such a perturbation results in an incomplete block in cell death [e.g., 2.6 extra VC-like cells in *ced-9(n3377); ced-3(+)* animals versus 4.7 extra cells in *ced-9(n3377); ced-3(2427)* animals (Fig. 2, B and C)]; by contrast, an *egl-1* null allele results in a near complete block in cell death (*12*). This difference indicates that EGL-1 interaction with CED-9 promotes cell death by doing more than simply disrupting the presumptive CED-9–CED-4 interaction.

**Fig. 6.**
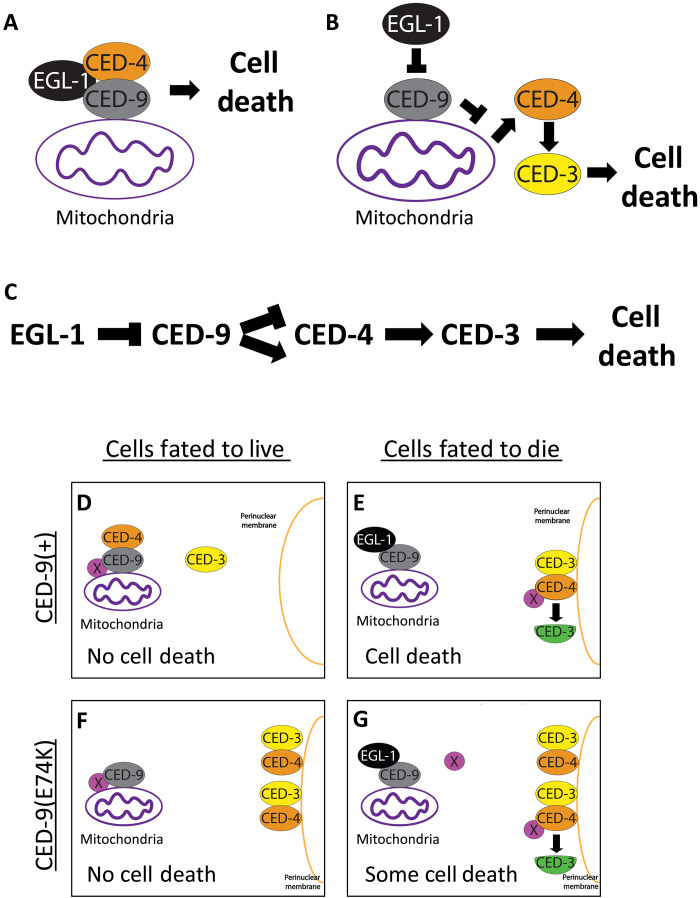
Possible alternatives to the canonical apoptosis pathway. (**A**) EGL-1 binding to the CED-9–CED-4 complex promotes a pro-apoptotic process dependent on a CED-9–CED-4 interaction. (**B**) EGL-1 blocks the ability of CED-9 to prevent a pro-apoptotic process occurring independently of CED-4 sequestration by CED-9. (**C**) Alternative genetic pathway in which CED-9 has both an anti-apoptotic and pro-apoptotic function that depend on CED-4 and CED-3. (**D** to **G**) An unknown (essential or genetically redundant) factor X promotes apoptosis through CED-4 activation. (D) In CED-9(+) cells fated to live, both factor X and CED-4 are sequestered to mitochondria by CED-9. (E) In CED-9(+) cells fated to die, EGL-1 changes the conformation of CED-9, releasing factor X–activated CED-4, which then translocates to the perinuclear membrane and initiates cell death. (F) CED-9–CED-4 binding mutants, such as *ced-9(n3377)* [E74K], maintain CED-9–dependent factor X sequestration, preventing killing in cells fated to live. (G) In cells fated to die carrying CED-9–CED-4 binding mutations, EGL-1 releases factor X from CED-9; CED-4 and factor X do not interact at mitochondria, leading to inefficient CED-4 activation by factor X and resulting in only some cell death.

In principle, the answers to these three questions might all involve a single unknown mechanism. Two nonmutually exclusive possibilities consistent with our data are as follows:

1) The binding of EGL-1 to the CED-9–CED-4 complex triggers a pro-apoptotic process (Fig. 6A). Loss of a CED-9–CED-4 interaction prevents this EGL-1–CED-9–CED-4 interaction and the pro-apoptotic process that it promotes.

2) The anti-apoptotic function of * ced-9* is to block a pro-apoptotic process dependent on CED-4 and CED-3 but independent of CED-4 sequestration to mitochondria by CED-9 (Fig. 6B).

A more specific model that incorporates both of these concepts is depicted in Fig. 6 (D to G): EGL-1 interaction with CED-9 activates a cell-death promoting factor or process (labeled “X”) that activates CED-4. CED-4 activation by X occurs more efficiently at mitochondria but can still occur, albeit less effectively, when CED-4 is not at mitochondria (such as in CED-9–CED-4 interaction mutants). X could reflect a pro-apoptotic factor from mitochondria that interacts with CED-4, a CED-4 conformational change, a chemical modification of CED-4, or CED-4 oligomerization. A model in which CED-9 sequesters a pro-apoptotic factor that activates CED-4 upon its release from mitochondria closely mirrors the mechanism of APAF-1 activation by mammalian BCL-2 family members via the release of cytochrome c from mitochondria (*28*–*32*).

It is also possible that CED-9 and CED-4 have two distinct physical interactions: one that is anti-apoptotic and one that is pro-apoptotic. In such a model, CED-4(R117S) and the CED-9 mutations that result in ectopic cell survival and viability disrupt the pro-apoptotic but not the anti-apoptotic interaction. In this model, the anti-apoptotic role of CED-9 is as in the canonical model and dependent on a CED-9–CED-4 interaction distinct from that defined by crystal structure studies to date (*21*), while the pro-apoptotic role of CED-9 depends on the CED-4 interaction defined by the crystal structure (*21*), which is distinct from the canonical, anti-apoptotic interaction. We tested one plausible model involving two distinct physical interactions in which the CED-9–CED-4 interaction mutants described above specifically perturb the CED-9 interaction with either the major and pro-apoptotic CED-4S or the minor and apparently anti-apoptotic CED-4L isoform of CED-4. However, we failed to identify any endogenous function for CED-4L, and the phenotype of animals retaining CED-4L and lacking CED-4S and that of animals lacking *ced-4* function altogether were no different (fig. S6). These data do not support a model in which the CED-9–CED-4 interaction mutants we describe differentially affect interactions of CED-9 with the two CED-4 isoforms.

In short, we report that an interaction between the BCL-2 homolog CED-9 and its pro-apoptotic binding partner the APAF-1 homolog CED-4 promotes apoptosis in *C. elegans*, the animal in which caspase-driven apoptosis was found. Like CED-9, the mammalian protein BCL-2 and other anti-apoptotic BCL-2 family members can be pro-apoptotic, as is the case for BCL-2 in the presence of the nuclear hormone receptor NUR77 (*33*). Despite extensive research spanning decades, the pro-apoptotic mechanism(s) of these bifunctional proteins remains poorly understood. One difficulty in elucidating such mechanisms might be the complexity of mammalian apoptosis pathways, a consequence in part of the relatively large number of BCL-2 family members in mammals. By contrast, CED-9 is the only BCL-2 family member in *C. elegans*. In addition, by using *C. elegans*, the functions of and interactions among cell-death proteins expressed at endogenous levels can be easily analyzed in the intact organism, features more difficult to attain using mammals or mammalian cell culture in vitro systems.

The pro-apoptotic function of *ced-9* is lost when a CED-9–CED-4 interaction is disrupted and CED-4 translocates away from mitochondria, suggesting that mitochondrial localization is important in promoting CED-4 activation and consequent apoptosis in *C. elegans*. In mammals, pro-apoptotic members of the BCL-2 family, such as BAX and BAK, promote apoptosis by forming pores in and permeabilizing the mitochondrial outer membrane and thereby releasing cytochrome c, which activates APAF-1, the mammalian homolog of *C. elegans* CED-4. *C. elegans* lacks such pore-forming pro-apoptotic BCL-2 family members, raising the question of why CED-4 activation might involve mitochondrial localization. Given the notable conservation of cell-death proteins and pathways between *C. elegans* and mammals and the apparently conserved role of mitochondria in CED-9/BCL-2–dependent CED-4/APAF-1 activation, the answer to this question might add a key insight concerning the fundamental process of apoptosis and suggest novel targets for therapeutics aimed at treating disorders that involve dysregulated apoptosis, such as certain neurodegenerative disorders and cancers.

## MATERIALS AND METHODS

### *C. elegans* strain maintenance

All strains were cultured on nematode growth medium (NGM) plates seeded with the *E. coli* strain OP50 at 20°C as described previously (*34*) unless otherwise indicated.

### Immunostaining

Embryos were collected by dissolving adult worms in bleach solution (0.5 M NaOH/0.8% sodium hypochlorite) for ~10 min (*35*). Embryos were then fixed and permeabilized essentially as described previously (*36*). Previously described rat antiserum against CED-4 was used for this study (*9*). We purified the rat antiserum against CED-4 using a Melon Gel IgG Purification Kit from Thermo Fisher Scientific (catalog no. 45206). Purified anti–CED-4 antibody was diluted to 10 μg/ml for immunofluorescence staining and then stained with a secondary antibody, goat anti-Rat IgG (H+L) Cross-Adsorbed Secondary Antibody, Alexa Fluor 647 from Thermo Fisher Scientific (catalog no. A-21247, RRID:AB_141778). Embryos were then mounted using a SlowFade Diamond Antifade Mountant with 4′,6-diamidino-2-phenylindole (DAPI) from Thermo Fisher Scientific (catalog no. S36964). Images of anti–CED-4 stained embryos were obtained using a 63x objective on an LSM 800 confocal microscope from Zeiss, Oberkochen, Germany (Zeiss LSM 800 with Airyscan Microscope, RRID:SCR_015963) and ZEN software. Images were obtained via confocal microscopy and, due to variability in the intensity of signal associated with in vivo immunofluorescence staining due to the freeze-cracking protocol used, channels (DAPI, MitoFluor, and CED-4) were processed individually using the Fiji software (Fiji, RRID:SCR_002285) (*37*). For examining mitochondria localization in antibody staining experiments, MitoFluor Red 589 from Thermo Fisher Scientific (catalog no. M-22424) was fed to adult worms at a concentration of 10 μg/ml by covering the lawn of OP50 and incubating overnight at 20°C essentially as described previously (*38*). Images were then cropped to reduce background. For examining mitochondrial localization in embryos carrying *wrmScarlet::ced-9*, MitoTracker Deep Red FM (Thermo Fisher Scientific, catalog no. M22426) was reconstituted in dimethyl sulfoxide (DMSO) (MilliporeSigma, product no. D8418) at a stock concentration of 1 mM. Twenty L4 worms were incubated overnight in the dark at 20°C on NGM petri plates seeded with *E. coli* OP50 containing either 5 μM MitoTracker Deep Red FM or 200X dilution of DMSO. Embryos were washed off the plates with M9 and imaged using confocal microscopy (Zeiss LSM 800 with Airyscan Microscope, RRID:SCR_015963) and ZEN software. Images were processed using the Fiji software (Fiji, RRID:SCR_002285) (*37*). Cartoon images were created using Adobe Illustrator (Adobe Illustrator, RRID:SCR_010279) and Microsoft PowerPoint.

### VC-like cell counts

The number of VC-like cells was assessed by counting the number of cells expressing the transgene *nIs106* in L4-stage worms (*n* = 50 for each genotype) using a Nikon SMZ18 fluorescent dissecting microscope (*20*). Counts were visualized using GraphPad Prism (RRID:SCR_002798).

### CRISPR-induced mutations

CRISPR mutants were generated by injecting early adult-stage worms carrying the transgene *nIs106* with Cas9 protein with a guide RNA (gRNA) targeting the gene *dpy-10* as well as a *dpy-10* repair template DNA and *ced-9* or *ced-4* repair template DNA (varying with each round of injection based on the gRNA) as described previously (*39*). *ced-9* mutations in the CED-4 binding pocket were generated by using target gRNAs against various locations in the CED-4 binding pocket based on the CED-9–CED-4 crystal structure (*21*); viable progeny showing extra VC-like cells were then picked and propagated. Structural images of the CED-9–CED-4 complex were generated based on the CED-9–CED-4 crystal structure using PyMOL (*21*, *40*). To create a *wrmScarlet::ced-9* knock-in strain, a polymerase chain reaction product containing sequences for *wrmScarlet* and a flexible linker 3x(Gly-Gly-Ser-Gly) was inserted just upstream of the start codon of *ced-9*. The DNA melting method was applied to prepare the repair template, as described previously (*41*).

### His-mediated pulldown assay

Truncated CED-9, CED-9[1-251], and full-length CED-4S protein were cloned as glutathione *S*-transferase (GST) fusion proteins into the vector pGEX-2T and as a 6x-His fusion protein into the vector pET-28b, respectively. CED-9 and CED-4 fusion proteins were overexpressed in the *E. coli* strain BL21(DE3) essentially as described previously (*22*). Competent cells were obtained from Thermo Fisher Scientific (catalog no. EC0114). *E. coli* strains coexpressing both plasmids were then pelleted, and pellets were resuspended in 5 ml of a lysis buffer [25 mM Tris (pH 8.0), 300 mM NaCl, 2 mM dithiothreitol (DTT), DNase I, and a Thermo Fisher Scientific Pierce Protease Inhibitor Tablet (catalog no. A32963)] and sonicated. The CED-4 protein was detected in these samples by Western blot using a Thermo Fisher Scientific 6x-His tag monoclonal antibody (catalog no. MA1-21315). The relative amount of CED-4 protein was determined using the Fiji software (Fiji, RRID:SCR_002285) (*37*), and 1 ml of the cell lysate normalized to the relative amount of CED-4 protein present was then allowed to bind with 60 μl of the Thermo Fisher Scientific HisPur Ni-NTA slurry (catalog no. 88221), which was washed twice with 500 μl of a wash buffer [25 mM Tris (pH 8.0), 300 mM NaCl, 2 mM DTT, and 25 mM imidazole (from 1 M stock at pH 8.0)] (*21*). The protein was allowed to incubate with the slurry at 4°C for 2 hours with rotation. The resin was then washed four times with 500 μl of the wash buffer and eluted with 45 μl of the Bio-Rad 4x Laemmli Sample Buffer (catalog no. 1610747) with DTT. Ten microliters of the sample was then resolved by SDS–polyacrylamide gel electrophoresis (PAGE) on a Bio-Rad (Hercules, CA) 10% Mini-PROTEAN TGX Precast Protein Gel (catalog no. 456-1034) and transferred to a Bio-Rad 0.45-μm nitrocellulose membrane (catalog no. 1620115) using a Thermo Fisher Scientific Pierce G2 Fast Blotter (catalog no. 62289). The CED-4 protein was visualized using a 1/2000 dilution of a Thermo Fisher Scientific 6x-His tag monoclonal antibody (catalog no. MA1-21315) and a 1/10,000 dilution of a Thermo Fisher Scientific Goat anti-Mouse IgG (H+L) Highly Cross-Adsorbed Secondary Antibody, Alexa Fluor Plus 647 (catalog no. A32728). The CED-9 protein was visualized using a 1/3000 dilution of a Thermo Fisher Scientific GST tag polyclonal antibody (catalog no. A5800) and a 1/10,000 dilution of Goat anti-Rabbit IgG (H+L) Highly Cross-Adsorbed Secondary Antibody, Alexa Fluor Plus 488 (catalog no. A32731).

### Western blot analysis

Protein was extracted from mixed-stage *C. elegans* embryos via 10 min of sonication at 80°C and 2 min of boiling at 95°C essentially as described previously (*42*). One hundred micrograms of the protein extracted from mixed-staged embryos was then resolved by SDS-PAGE on a Bio-Rad (Hercules, CA) 10% Mini-PROTEAN TGX Precast Protein Gel (catalog no. 456-1034) and transferred to a Bio-Rad 0.45-μm nitrocellulose membrane (catalog no. 1620115) using a Thermo Fisher Scientific Pierce G2 Fast Blotter (catalog no. 62289). The CED-4 protein was detected using the previously described purified rat anti–CED-4 antibody at a concentration of 12 μg/ml, and the secondary antibody, goat anti-Rat IgG (H+L) Cross-Adsorbed Secondary Antibody, Alexa Fluor 647 from Thermo Fisher Scientific, (catalog no. A-21247, RRID:AB_141778) was used at a concentration of 2 μg/ml essentially as described previously (*42*). Protein band size was determined using Bio-Rad Precision Plus Protein Dual Color Standards (catalog no. 1610374).
