# Supplementary material for: The pro-apoptotic function of the C. elegans BCL-2 homolog CED-9 requires interaction with the APAF-1 homolog CED-4
Source: Sci Adv. 2024 Oct 9;10(41):eadn0325. doi: 10.1126/sciadv.adn0325 (PMC11817491; doi:10.1126/sciadv.adn0325)
Supplement: Supplementary file 1 — Tables S1 to S4 Figs. S1 to S6 [file sciadv.adn0325_sm.pdf]

Science Advances
AAAS

Supplementary Materials for

The pro-apoptotic function of the *C. elegans* BCL-2 homolog CED-9 requires interaction with the APAF-1 homolog CED-4

Nolan Tucker *et al.*

Corresponding author: H. Robert Horvitz, horvitz@mit.edu

Sci. Adv. **10**, eadn0325 (2024)
DOI: 10.1126/sciadv.adn0325

This PDF file includes:

Tables S1 to S4
Figs. S1 to S6

Strain	% Animals with an ectopic M4-like cell (*$P_{ceh\text{-}28}$::mCherry*)	% Animals with at least one ectopic RIM/RIC-like cell (*$P_{tdc\text{-}1}$::GFP*)
WT	0	0
ced-3(n2427)	56	50
ced-9(n2812); ced-3(n2427)	98	100
ced-9(n3377)	37	72
ced-4(n6703)	26	28

Table S1. Loss-of the pro-apoptotic function of CED-9 causes ectopic cell-survival of multiple neuronal types. An extra M4-like cell is observed in 37% of animals carrying *ced-9(n3377)* and 26% of animals carrying *ced-4(n6703)* (n=50). At least one additional RIM/RIC-like cell is observed in 72% of animals carrying *ced-9(n3377)* and 28% of animals carrying *ced-4(n6703)* (n=50).

	% Hatching (n=100)			
Allele	15°C	20°C	25°C	% Animals with at least one ectopic VC-cell death (***$P_{lin\text{-}11}$::GFP***) (n=100) (20°C)
ced-9(n2812)	0	0	0	98
ced-9(n3377)	100	100	100	0
ced-9(n6697)	100	100	100	0
ced-9(n6698)	100	100	100	0
ced-9(n6704)	100	100	100	0
ced-9(n6705)	100	100	100	0
ced-9(n6712)	100	100	98	0
ced-9(n6730)	100	96	94	0
ced-4(n6703)	100	100	100	0
ced-4(+) ced-9(+)	100	100	100	0

Table S2. CED-9-CED-4 binding mutants retain the anti-apoptotic function of CED-9. The CED-9-CED-4 binding mutant alleles result in viability when homozygous and thus fail to cause the maternal-effect lethality typical of *ced-9* null alleles. n=100 embryos were observed at 15°C, 20°C, and 25°C and % hatching was recorded for *ced-9* and *ced-4* alleles with no mutations in any other cell-killing gene, such as *ced-3*. Unlike the *ced-9* null allele *n2812*, these alleles do not cause ectopic deaths of VC cells (n=100 animals).

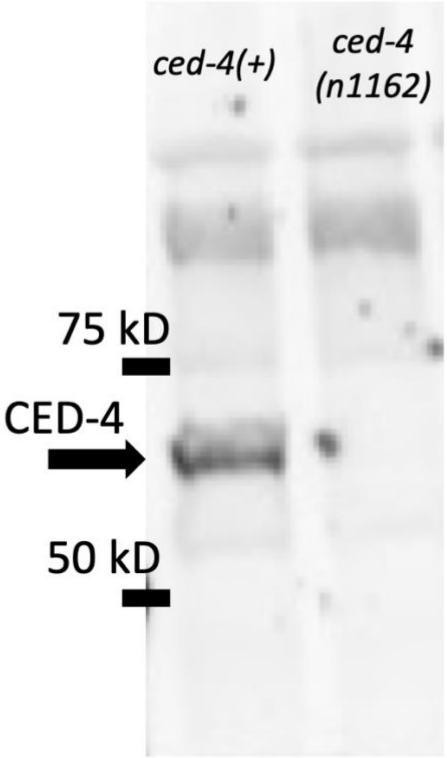

Fig. S1. CED-4 protein is detected by anti-CED-4 antibody. A western blot showing that a polyclonal antibody made against CED-4 protein identifies a band between 50 kD and 75 kD (see Methods), roughly the size of CED-4 protein (63 kD), in a lane containing protein extracted from mixed-staged embryos carrying a wild-type *ced-4* allele. The antibody failed to detect this band in embryos carrying the null allele *ced-4(n1162).* These two images were cropped from a larger image.

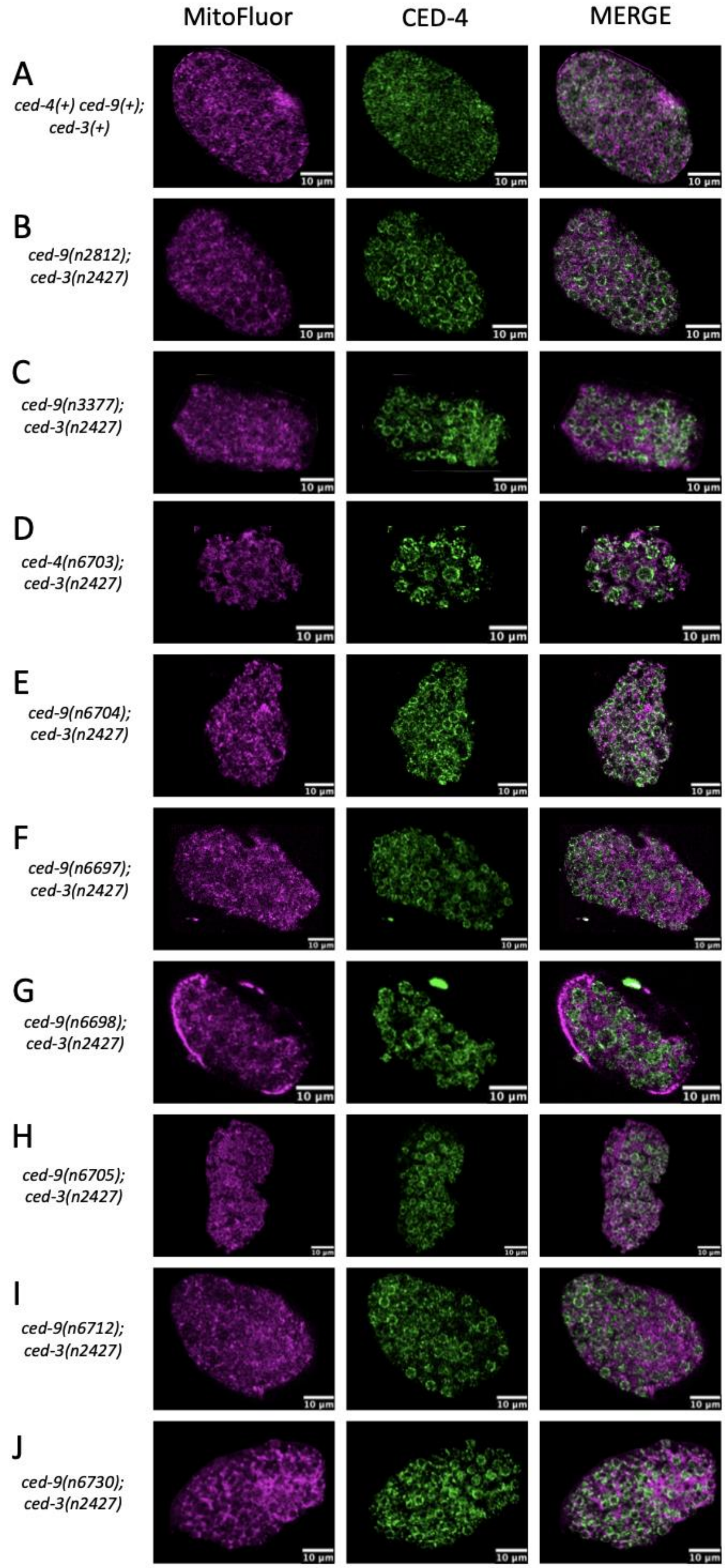

MitoFluor
CED-4
MERGE
A
ced-4(+) ced-9(+); ced-3(+)
B
ced-9(n2812); ced-3(n2427)
C
ced-9(n3377); ced-3(n2427)
D
ced-4(n6703); ced-3(n2427)
E
ced-9(n6704); ced-3(n2427)
F
ced-9(n6697); ced-3(n2427)
G
ced-9(n6698); ced-3(n2427)
H
ced-9(n6705); ced-3(n2427)
I
ced-9(n6712); ced-3(n2427)
J
ced-9(n6730); ced-3(n2427)
10 µm

Fig. S2. Mutations that disrupt the CED-9-CED-4 binding regions of either CED-9 or CED-4 disrupt sequestration of CED-4 to mitochondria. A) Mitochondrial localized CED-4 in wild-type embryos, **B)** perinuclear localization of CED-4 in *ced-9(n2812); ced-4(n2427)* embryos, **C)** perinuclear localization of CED-4 in *ced-9(n3377); ced-4(n2427)* embryos, **D)** perinuclear localization of CED-4 in *ced-4(n6703); ced-4(n2427)* embryos, **E)** perinuclear localization of CED-4 in *ced-9(n6704); ced-4(n2427)* embryos**, F)** perinuclear localization of CED-4 in *ced-9(n6697); ced-4(n2427)* embryos, **G)** perinuclear localization of CED-4 in *ced-9(n6698); ced-4(n2427)* embryos, **H)** perinuclear localization of CED-4 in *ced-9(n6705); ced-4(n2427)* embryos**, I)** perinuclear localization of CED-4 in *ced-9(n6712); ced-4(n2427)* embryos, and **J)** perinuclear localization of CED-4 in *ced-9(n6730); ced-4(n2427)* embryos. Images were cropped to avoid background and the brightness and contrast of the MitoFluor and CED-4 channels were adjusted individually using Fiji software.

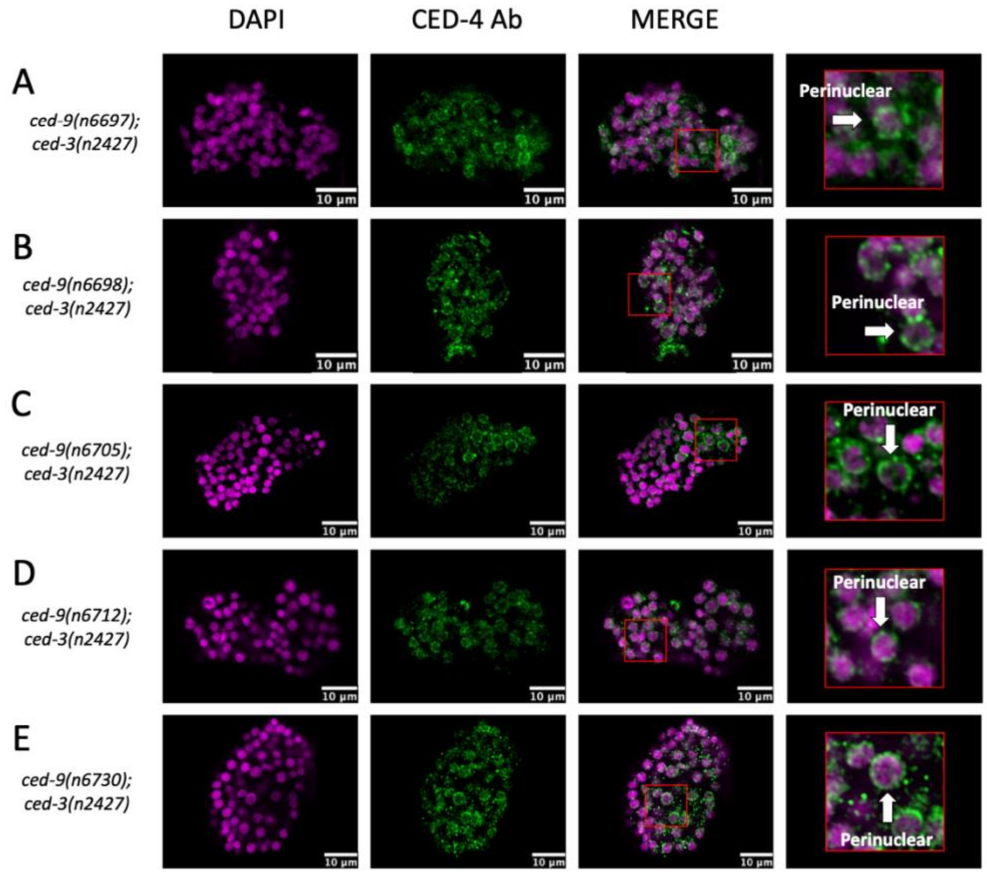

Fig. S3. Mutations that disrupt the CED-9-CED-4 binding regions of either CED-9 or CED-4 result in the mislocalization of CED-4 to the perinuclear membrane. A) Perinuclear localization of CED-4 in *ced-9(n6697); ced-4(n2427)* embryos. **B)** Perinuclear localization of CED-4 in *ced-9(n6698); ced-4(n2427)* embryos. **C)** Perinuclear localization of CED-4 in *ced-9(n6705); ced-4(n2427)* embryos. **D)** Perinuclear localization of CED-4 in *ced-9(n6712); ced-4(n2427)* embryos. **E)** Perinuclear localization of CED-4 in *ced-9(n6730); ced-4(n2427)* embryos. Images were cropped to avoid background and the brightness and contrast of the DAPI and CED-4 channels were adjusted individually using Fiji software.

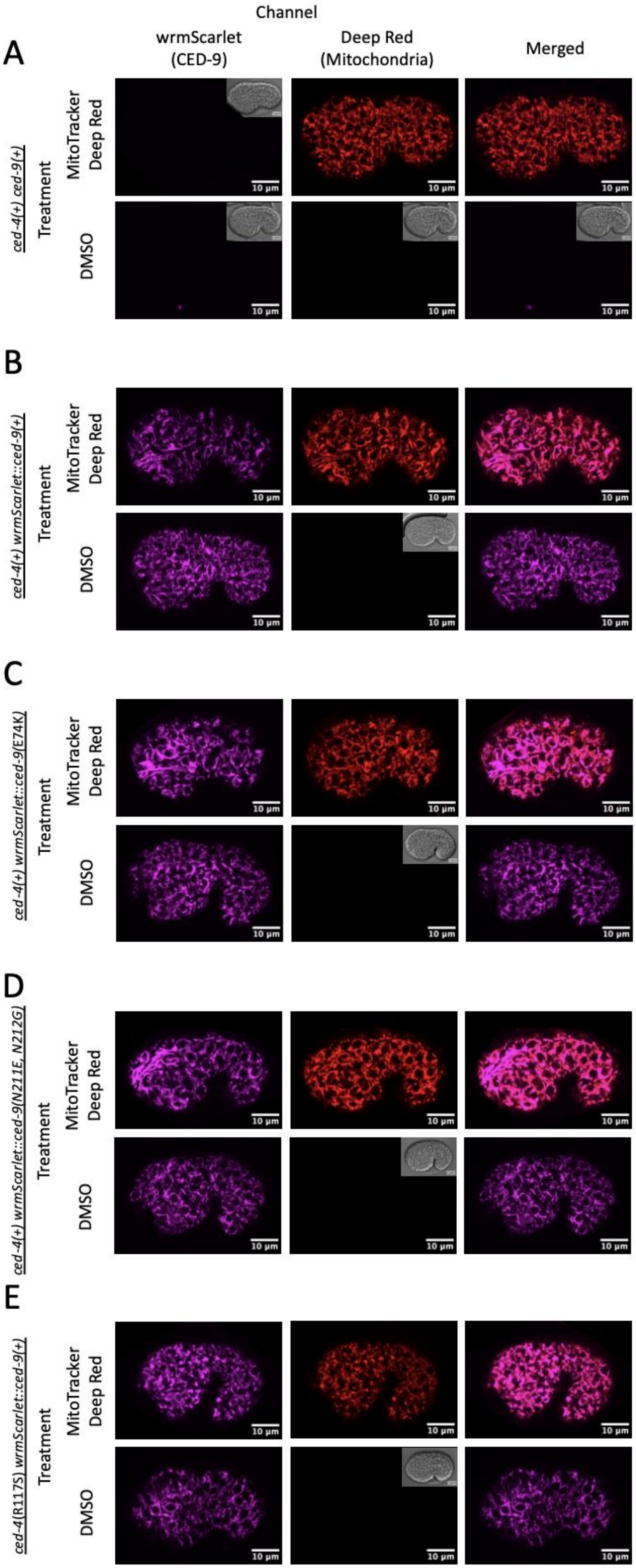
Channel
wrmScarlet (CED-9)
Deep Red (Mitochondria)
Merged
A
ced-4(+) ced-9(+)
Treatment
MitoTracker Deep Red
DMSO
B
ced-4(+) wrmScarlet::ced-9(+)
C
ced-4(+) wrmScarlet::ced-9(E74K)
D
ced-4(+) wrmScarlet::ced-9(N211E, N212G)
E
ced-4(R117S) wrmScarlet::ced-9(+)
10 µm

Fig. S4. CED-9-CED-4 interaction mutations do not disrupt the localization of CED-9 to mitochondria. To determine whether CED-9-CED-4 interaction mutants disrupt the localization of CED-9 to mitochondria, we treated worms with MitoTracker Deep Red or DMSO and assessed the localization pattern of CED-9 in embryos using alleles of CED-9 tagged with the red fluorescent protein wrmScarlet at the N-terminus of CED-9 and expressed from the endogenous *ced-9* locus. The tagged *wmScarlet::ced-9(+)* allele is *n6731,* and tagged versions of mutant *ced-9* alleles are all double mutants with *n6731.* **A)** Localization of mitochondria in wild-type embryos, which lack wmScarlet::CED-9. **B)** Mitochondrial localization of CED-9 in *ced-4(+) wrmScarlet::ced-9(+)* embryos (*ced-9(n6731))*. **C)** Mitochondrial localization of CED-9 in *ced-4(+) wrmScarlet::ced-9*(E74K) embryos (*ced-9(n6731 n6806))*. *ced-9(n6806)* carries the missense mutation E74K. **D)** Mitochondrial localization of CED-9 in *ced-4(+) wrmScarlet::ced-9*(N211E, N212G) embryos (*ced-9(n6731 n6805))*. *ced-9(n6805)* carries two missense mutations, R211E and N212G. **E)** Mitochondrial localization of CED-9 in *ced-4*(R117S) *wrmScarlet::ced-9*(+) embryos *(ced-4(n6703) ced-9(n6731))*. *ced-4(n6703)* carries the missense mutation R117S. Images were cropped to avoid background, and the brightness and contrast of the Brightfield, wrmScarlet, and MitoTracker Deep Red channels were adjusted individually using Fiji software. *nIs106* is present in the background of all strains for consistency and was used to confirm the presence of a cell-death phenotype.

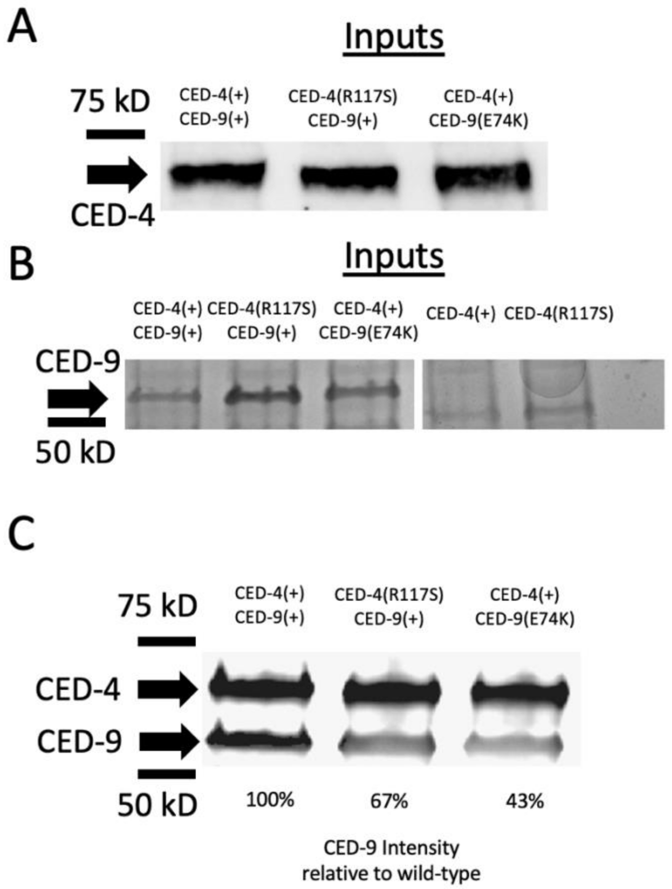

Fig. S5. CED-9(E74K) and CED-4(R117S) are deficient in CED-9-CED-4 complex formation *in vitro*. Western blot analysis of interactions between wild-type and mutant truncated CED-9 and wild-type and mutant CED-4 proteins. CED-9 and CED-4 proteins were GST and 6x-His tagged, respectively, and co-expressed in the *E. coli* strain BL21(DE3). CED-9(+) and CED-9(E74K) were expressed by the plasmids pNT004 and pNT006, respectively. CED-4(+) and CED-4(R117S) were expressed by the plasmids pNT007 and pNT008, respectively. CED-4 protein was detected in co-expressed cell lysates by a 6x-His tag monoclonal antibody, the lysates were then adjusted to contain comparable amounts of CED-4 protein. **A)** Western blot showing CED-4 protein from lysate aliquots adjusted to contain comparable levels of CED-4 protein, detected using a 6x-His tag monoclonal antibody. **B)** Image of a region of a Coomassie-stained SDS-PAGE gel showing the levels CED-9 present in the samples shown in panel (A) and

used for the Western blots shown in panel (C). The 57 kD GST-tagged truncated CED-9 band was not present in two lanes (cropped from the same gel) containing samples prepared from cell lysates that lacked CED-9-expressing plasmids (see Methods). **C)** A western blot showing CED-4 and CED-9 proteins detected using a 6x-His tag monoclonal and a GST tag polyclonal antibody, respectively, after lysates containing approximately equal levels of CED-4 protein (as shown in panel (A)) were mixed with a Ni-NTA resin.

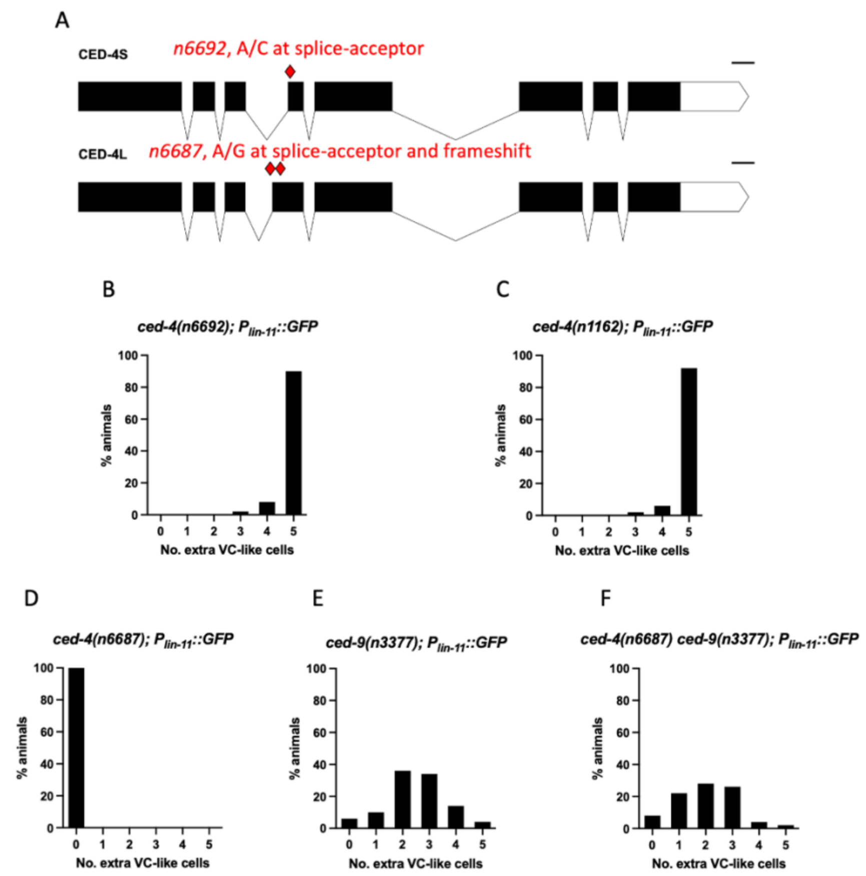

G

Allele	% Hatching (n=100) 15°C	20°C	25°C	% Animals with at least one ectopic VC-cell death (*Plin-11::GFP*) (n=100) (20°C)
ced-9(n2812)	0	0	0	98
ced-4(n6692)	100	100	100	0
ced-4(n6687)	100	100	100	0
ced-4(n1162)	100	100	100	0
ced-4(+) ced-9(+)	100	100	100	0

Fig. S6. Loss-of the long-isoform of CED-4 causes no detectable cell-killing defect. A) To test the effects of the two presumptive CED-4 isoforms– short (CED-4S) and long (CED-4L) – we used CRISPR to generate CED-4L- and CED-4S-specific alleles of *ced-4.* The CED-4L-specific allele, *ced-4(n6692),* lacks CED-4S and retains CED-4L since this allele contains a CED-4S-specific splice-acceptor site mutation that also results in a sense-mutation (Arg-to-Arg) in CED-4L protein. The CED-4S-specific allele, *ced-4(n6687)*, lacks CED-4L and retains CED-4S, since this allele contains a CED-4L-specific splice-acceptor site mutation and causes a 2-nt

insertion in the CED-4L-specific coding region resulting in a frameshift mutation. **B-C)** *ced-4(n6692)* (lacking CED-4S and retaining CED-4L) causes a cell-killing defect similar to that of a null allele of *ced-4, n1162.* **D-F)** *ced-4(n6687)* (lacking CED-4L and retaining CED-4S) shows no detectable cell-death defect on its own, nor does it suppress the cell-death defect caused by *ced-9(n3377).* **G)** *ced-4(n6692)* and *ced-4(n6687)* mutants are viable as homozygotes, lacking maternal-effect lethality. n=100 embryos were observed at 15°C, 20°C, and 25°C and % hatching was recorded for *ced-4* alleles with no mutations in any other cell-killing gene, e.g. *ced-3*. Unlike the *ced-9* null allele *n2812*, these alleles do not cause ectopic deaths of VC cells (n=100 animals).

Strain	Source	Strain number
ced-4(n1162) unc-69(e587) ced-9(n1950 n2161)	Michael Hengartner / Horvitz lab collection	MT5613
unc-69(e587) ced-9(n1950 n2161); ced-3(n2427)	Michael Hengartner / Horvitz lab collection	MT6259
ced-4(n2860) unc-69(e587) ced-9(n1950 n2161)	Horvitz lab collection	MT7667
ced-4(n2879) unc-69(e587) ced-9(n1950 n2161)	Horvitz lab collection	MT7746
nIs106[P_{lin-11}::GFP]	Nancy Tsung / Scott Cameron / Horvitz lab collection	MT9970
ced-4(n2879); nIs106[P_{lin-11}::GFP]	Fangli Chen / Barbara Conradt / Horvitz lab collection	MT10031
ced-3(n2427); nIs106[P_{lin-11}::GFP]	Peter Reddien / Horvitz lab collection	MT10113
ced-3(n2427); nIs106[P_{lin-11}::GFP]	Peter Reddien / Horvitz lab collection	MT10113
ced-4(n1162); nIs106[P_{lin-11}::GFP]	Scott Cameron / Horvitz lab collection	MT10332
ced-4(n3141); nIs106[P_{lin-11}::GFP]	Brad Hersh / Horvitz lab collection	MT10486
ced-4(n3141) unc-69(e587) ced-9(n1950 n2161)	Brad Hersh / Horvitz lab collection	MT11158
ced-3(n3692); nIs106[P_{lin-11}::GFP]	Bradley Hersh / Horvitz lab collection	MT12054
ced-9(n2812); ced-3(n2427);; nIs106[P_{lin-11}::GFP]	Peter Reddien / Horvitz lab collection	MT12659
ced-9(n3377); ced-3(n2427); ; nIs106[P_{lin-11}::GFP]	Peter Reddien / Horvitz lab collection	MT12661
ced-9(n3377); nIs106[P_{lin-11}::GFP]	Peter Reddien / Horvitz lab collection	MT12727
nIs180[P_{tdc1}::GFP]; ced-3(n2427)	Daniel Denning / Horvitz lab collection	MT18148
ced-4(n2860); nIs106[P_{lin-11}::GFP]	Horvitz lab collection	MT18967
ced-4(n3392 n5611); nIs106[P_{lin-11}::GFP]	Daniel Denning / Peter Reddien / Horvitz lab collection	MT21407
ced-9(n6676); nIs106[P_{lin-11}::GFP]	This study	MT26819
ced-4(n6687); nIs106[P_{lin-11}::GFP]	This study	MT26954
ced-4(n6692); nIs106[P_{lin-11}::GFP]	This study	MT26959
ced-4(n6687); ced-9(n3377); nIs106[P_{lin-11}::GFP]	This study	MT26960
ced-9(n6697); nIs106[P_{lin-11}::GFP]	This study	MT27040
ced-9(n6698); nIs106[P_{lin-11}::GFP]	This study	MT27106
ced-9(n6705); nIs106[P_{lin-11}::GFP]	This study	MT27137
ced-9(n6704); nIs106[P_{lin-11}::GFP]	This study	MT27138
ced-4(n6703); nIs106[P_{lin-11}::GFP]	This study	MT27143
ced-9(n6712); nIs106[P_{lin-11}::GFP]	This study	MT27156
ced-9(n6715); nIs106[P_{lin-11}::GFP]	This study	MT27159
ced-4(n6703); ced-3(n2427); nIs106[P_{lin-11}::GFP]	This study	MT27247
ced-9(n6704); ced-3(n2427); nIs106[P_{lin-11}::GFP]	This study	MT27248
ced-9(n6697); ced-3(n2427); nIs106[P_{lin-11}::GFP]	This study	MT27249
ced-9(n6705); ced-3(n2427); nIs106[P_{lin-11}::GFP]	This study	MT27264
ced-9(n6712); ced-3(n2427); nIs106[P_{lin-11}::GFP]	This study	MT27266
ced-9(n6698); ced-3(n2427); nIs106[P_{lin-11}::GFP]	This study	MT27267
ced-4(n6703) unc-69(e587am) ced-9(n1950dm) ced-9(n2161) / qC1 III [dpy-19(e1259ts,mat) glp-1(q339)] nIs189[P_{myo-2}::GFP]]; nIs106[P_{lin-11}::GFP]	This study	MT27292
ced-4(n6703); nIs348[P_{ced-28}::mCherry]; nIs106[P_{lin-11}::GFP]	This study	MT27367
ced-9(n6730); nIs106[P_{lin-11}::GFP]	This study	MT27368
ced-9(n3377); nIs348[P_{ced-28}::mCherry]; nIs106[P_{lin-11}::GFP]	This study	MT27377
ced-9(n6730); ced-3(n2427); nIs106[P_{lin-11}::GFP]	This study	MT27378
ced-9(n6731 [wrmScarlet::ced-9])	This study	MT27380
ced-9(n2812) / qC1 III [dpy-19(e1259ts,mat) glp-1(q339) nIs189[P_{myo-2}::GFP]] ; nIs106[P_{lin-11}::GFP]	This study	MT27471
nIs180[P_{tdc1}::GFP]; ced-4(n6703)	This study	MT27472
ced-9(n3377); ced-3(n3692); nIs106[P_{lin-11}::GFP]	This study	MT27473
nIs180[P_{tdc1}::GFP]; ced-9(n3377)	This study	MT27474
nIs180[P_{tdc1}::GFP]; ced-9(n2812); ced-3(n2427)	This study	MT27475
ced-9(n2812); ced-3(n2427); nIs348 P_{ced-28}::mCherry]; nIs106[P_{lin-11}::GFP]	This study	MT27511
ced-3(n2427); nIs348[P_{ced-28}::mCherry]; nIs106[P_{lin-11}::GFP]	This study	MT27512
ced-9(n6731 [wrmScarlet::ced-9])I; nIs106[P_{lin-11}::GFP]	This study	MT27626
ced-4(n6703) ced-9(n6731 [wrmScarlet::ced-9]); nIs106[P_{lin-11}::GFP]	This study	MT27644
ced-9(n6731 [wrmScarlet::ced-9] n6805); nIs106[P_{lin-11}::GFP]	This study	MT27645
ced-9(n6731 [wrmScarlet::ced-9] n6806); nIs106[P_{lin-11}::GFP]	This study	MT27647

Table S3. Key strain table.

Recombinant DNA	Source
CED-9(E74K) donor: GTCAATTGATGGAAAAATCAATGATTGGAAAGAGCCAAGGCTTGATATCGAAGGATTTGTGGTAATTTT TTAATTTTTTTTTGTAAATAAAATTTCCTGC	IDT DNA
CED-4(R117S) donor: TTTCCCGACAAATGCTCGATAGTAAACTATTGCTTGGAAATGTTCCAAAACAAATGACATGCTATATTCGA GAGTATCACGTGGATCGAGTGATCAAAAA	IDT DNA
CED-9(R211E, N212G) donor: GCAGGGACAAGTGCGAAACCTCTTCGTTTACACATCGCTGTTCATCAAAACGCGGATCGAGGGCAACT GGAAGGAACACAATCGGAGCTGGGTAAGGAGT	IDT DNA
dpy-10 donor: CACTTGAACTTCAATACGGCAAGATGAGAATGACTGGAAACCGTACCGCATGCGGTGCCTAGGTAGCG GAGCTTCACATGGCTTCAGACCAACAGCCTAT	IDT DNA
CED-9(E74K) donor: GTCAATTGATGGAAAAATCAATGATTGGAAAGAGCCAAGGCTTGATATCGAAGGATTTGTGGTAATTTT TTAATTTTTTTTTGTAAATAAAATTTCCTGC	IDT DNA
ced-4(n6687) donor: GAATGCGAGTCATCCGTGTCGCTCACAACACGGCCCGTCTTCAAACACACAAAACATGCAAGCGTCAC GAAATATCAAATTAAAAGCTAACCAGGTCAA	IDT DNA
ced-4(n6692) donor: ATGACTCGCATTCGATCACCGACTTCATTAACCGTGTTCTTTCACGAAGCGAAGACGATCTTCTCAATTTC CCATCGGTGGAGCATGTCACGTCAGTTGTACTCAAAAGG	IDT DNA
wrmScarlet::CED-9 donor: ATGGTCAGCAAGGGAGAGGCAGTTATCAAGGAGTTCATGCGTTTCAAGGTCCACATGGAGGGATCCAT GAACGGACACGAGTTCGAGATCGAGGGAGAGGGAGAGGGACGTCCATACGAGGGAACCCAAACCGC CAAGCTCAAGGTCACCAAGGGAGGACCACTCCCATTCTCCTGGGACATCCTCTCCCCACAATTCATGTAC GGATCCCGTGCCTTCATCAAGCACCCAGCCGACATCCCAGACTACTACAAGCAATCCTTCCCAGAGGGA TTCAAGTGGGAGCGTGTCATGAACTTCGAGGACGGAGGAGCCGTCACCGTCACCCAAGACACCTCCCT CGAGGACGGAACCCTCATCTACAAGGTCAAGCTCCGTGGAACCAACTTCCCACCAGACGGACCAGTCA TGCAAAAGAAGACCATGGGATGGGAGGCCTCCACCGAGCGTCTCTACCCAGAGGACGGAGTCCTCAA GGGAGACATCAAGATGGCCCTCCGTCTCAAGGACGGAGGACGTTACCTCGCCGACTTCAAGACCACCT ACAAGGCCAAGAAGCCAGTCCAAATGCCAGGAGCCTACAACGTCGACCGTAAGCTCGACATCACCTCC CACAACGAGGACTACACCGTCGTCGAGCAATACGAGCGTTCCGAGGGACGTCACTCCACCGGAGGAAT GGACGAGCTCTACAAGGGAGGATCTGGAGGCGGTTCTGGCGGAGGTTCTGGT	IDT DNA
**gRNA**	**Source**
E74K gRNA: AGAGCCAAGGCTTGATATCG	IDT DNA
R117S gRNA: TCGATAGGAAACTATTGCTT	IDT DNA
CED-9(R211E, N212G) gRNA: TGTGTTCCTTCCAGTTGTTG	IDT DNA
CED-9 gRNA: AACACAATCGGAGCTGGGTA	IDT DNA
dpy-10 gRNA: GCUACCAUAGGCACCACGAG	IDT DNA
ced-4(n6687) gRNA: ATCCGTGTCGCTCACAACAC	IDT DNA
ced-4(n6692) gRNA: CGATCTTCTCAATTTCCCAT	IDT DNA
wrmScarlet::CED-9 gRNA: ATTTTAGATGACACGCTGCA	IDT DNA

Table S4. Oligonucleotides used to construct CRISPR strains.
